# Inflammation-based prognostic scores and nutritional prognostic index in patients with locally-advanced unresectable colorectal cancer

**DOI:** 10.1186/1477-7819-12-210

**Published:** 2014-07-15

**Authors:** Masahide Ikeguchi, Sho-ichi Urushibara, Ryugo Shimoda, Manabu Yamamoto, Yoshihiko Maeta, Keigo Ashida

**Affiliations:** 1 Department of Surgery, Division of Surgical Oncology, Faculty of Medicine, Tottori University, Yonago 683-8504, Japan

**Keywords:** Chemotherapy, Colorectal cancer, Inflammation, Immunity, Nutrition

## Abstract

**Background:**

Unresectable colorectal cancer has a poor prognosis. However, some patients survive intensive chemotherapy, and complete resection of primary and metastatic tumors may even be possible. In the present study, we examined the prognostic factors associated with survival after intensive chemotherapy in patients with unresectable colorectal cancer.

**Methods:**

This retrospective study enrolled 61 patients diagnosed with unresectable locally advanced colorectal cancer between January 2004 and December 2013. Among the prognostic parameters, we found that the prognoses of patients with abnormal performance status (PS) of 2 or 3, high Glasgow Prognostic Score (GPS) of 1 or 2, high neutrophil/lymphocyte ratio (NLR) >5, and low prognostic nutritional index (PNI) <40 were poor. Thus, we scored each patient according to our scoring system (abnormal PS, 2 or 3 = +1; high GPS, 1 or 2 = +1; high NLR, >5 = +1; and low PNI, <40 = +1). If the patient showed abnormalities in every parameter, the score would be +4.

**Results:**

Sixteen patients had a score of 0, 17 scored +1, 10 scored +2, 17 scored +3, and one scored +4. The median survival time (MST) of the 61 patients was 9 months. Patients were divided into two groups, a low-score group (0 and +1) and a high-score group (+2, +3, and +4). The MST of the 33 patients in the low-score group was significantly longer than that of the 28 patients in the high-score group (15 months versus 4 months, *P* < 0.001). Also, conversion chemotherapy was performed in 4.9% (3/61) of patients. And these 3 patients were in a low-score group.

**Conclusions:**

This new prognostic scoring system may help to select patients with unresectable advanced colorectal cancer who are able to survive through intensive chemotherapy.

## Background

Unresectable locally advanced colorectal cancer has a poor prognosis. Many patients suffer from obstructive ileus or sub-ileus, and bypass surgery or artificial anus reconstruction may be needed to improve their quality of life. 5-Fluorouracil in combination with leucovorin plus oxaliplatin or irinotecan, with or without molecularly targeted drugs, has been administered as first-line and as the standard treatment for patients with unresectable colorectal cancer [1,2]. Many patients fail to recover from such palliative surgery and effective chemotherapy is therefore not possible. However, some patients undergo complete resection of primary and metastatic cancer sites after intensive chemotherapy, and may subsequently survive for long periods. It is therefore important to identify those patients who are able to survive through intensive chemotherapy.

Eastern Cooperative Oncology Group Performance Status (ECOG-PS) has been recognized as a good indicator for the use of intensive chemotherapy in advanced carcinoma [3,4]; however, this parameter lacks objectivity. The Glasgow Prognostic Score (GPS) has been shown to improve the accuracy of predicting cancer-specific survival in a variety of common solid tumors, including colorectal cancer [5,6]. Regarding measurement of the systemic inflammatory response, the combination of C-reactive protein (CRP) and albumin (ALB) may be useful for diagnosing not only chronic inflammation but also nutritional status in cancer patients. Chua and colleagues [7] and Yamanaka and colleagues [8] concluded that the neutrophil/lymphocyte ratio (NLR) in the peripheral blood of patients was one aspect of the chronic systemic inflammatory response that influenced clinical outcomes in patients with colorectal cancer, while Ubukata and colleagues [9] reported that elevated NLR indicated an immunosuppressive state in cancer patients. NLR in peripheral blood may thus be considered as both a marker of inflammation and also an immunological marker. Preoperative assessment of a patient’s immunological and nutritional conditions (prognostic nutritional index, or PNI) has recently been reported as an important marker in cancer patients [10,11]. Furthermore, serum carcinoembryonic antigen (CEA) level is known to be a good indicator for progression of colorectal cancer [12].

In the present study, we evaluated the objective prognostic markers GPS, NLR, PNI, and CEA in consecutive patients with unresectable advanced colorectal cancer and investigated whether these markers predicted response to chemotherapy and long-term survival.

## Methods

### Patients

A total of 615 primary colorectal cancer patients were introduced to our department for medical treatment between January 2004 and December 2013. Primary colorectal regions had been resected in 550 patients (89.4%), but colorectal resection had been abandoned in the remaining 65 patients (10.6%) because of advancement of primary or metastatic tumors, poor performance status (PS), or advanced age. Of these 65 patients, four with no advancement of tumors who refused further treatment were excluded from this study. Sixty-one patients with locally advanced colorectal cancer with invasion to neighboring important organs (such as the liver, pancreas, kidney, bladder, or sacral bone) with or without synchronous distant metastasis were diagnosed as initially unresectable. Local invasion of tumors were detected to cavitas pelvis including sacral bone in 19, to liver, duodenum, and pancreas in 11, to uterus or bladder in 18, and to kidney or ureter in 13 patients. Other clinicopathological characteristics of the 61 patients are shown in Table 1. Obstruction was the most commonly detected primary tumor-related symptom (obstruction, 30; bleeding or anemia, 5; obstruction and bleeding, 6; and local pain, 4). The 36 patients (59%) with obstruction caused by primary tumors underwent bypass surgery (n = 8), artificial anus reconstruction (n = 26), or both (n = 2). Metastases were found in 46 patients (75.4%), with liver metastasis the most frequently detected. No distant metastases were found in 15 patients.

**Table 1:** Clinicopathological characteristics of 61 patients

	
Age (years, mean ± SD (median and range))	67.8 ± 13.5 (68, 35-94)
Gender (male/female)	35/26
Eastern Cooperative Oncology Group Performance Status (0/1/2/3)	33/13/12/3
Location (colon/rectum)	32/29
Primary tumor-related obstruction (yes/no)	36/25
Metastasis (n)	
Liver only	20
Liver and lung	10
Liver and peritoneum	5
Extended lymph node only	4
Peritoneum only	4
Lung only	1
Bone only	1
Lung and bone	1
Treatment (n)	
Chemotherapy only	16
Surgical intervention + chemotherapy	25
Surgical intervention only	13
Best supportive care	7

Chemotherapy and chemo-radiotherapy with or without surgical intervention were performed in 41 patients (67.2%; chemotherapy only, n = 32; and chemo-radiotherapy, n = 9), but no effective treatment other than surgery was possible in a further 13 patients. Seven patients with advanced age or poor PS received best supportive care. The 61 patients were followed until May 2014 and the median follow-up time was 8 months.

### Blood samples

Blood samples were taken from each patient routinely at their first visit to our hospital. CRP, serum ALB, NLR, PNI, and CEA levels were analyzed for each patient.

### Scoring system

Patients were scored according to the original GPS. The GPS consists of the combination of CRP and ALB measurements. Patients with normal CRP (≤1.0 mg/dL) and normal ALB (≥3.5 g/dL) had a GPS of 0 and were classified as the low GPS group. Patients with either one abnormal factor (GPS = 1), or both abnormal factors (GPS = 2) were classified as the high GPS group. According to a previous report [7], an NLR ≥5 was considered abnormal. The PNI was calculated using the following formula: 10 × serum ALB concentration (g/dL) + 0.005 × lymphocyte count (number/mm^2^) in peripheral blood. The PNI cut-off value was determined to be 40 [10]. According to Hsu and colleagues [12], CEA ≥10 ng/mL was considered abnormal. All investigations were conducted in conformity with the Recommendations from the Declaration of Helsinki. Informed consent was obtained from all these 61 patients about their treatment. The study protocol has been approved by the ethics committee of Tottori University (approval number: 1223).

### Statistical analysis

Differences between two parameters were compared using χ^2^ tests for independence, Fisher’s exact probability test, and the Mann–Whitney *U* test. Spearman’s rank correlation coefficient was used to assess the correlation between two parameters. Survival rates were estimated by the Kaplan-Meier method, and the significance of differences between survival curves was examined by log-rank tests. A *P* value <0.05 was regarded as statistically significant.

## Results

The median survival time (MST) of the 61 patients was 9 months (range 1 to 75 months). Chemotherapy and chemo-radiotherapy were performed in 41 patients; however, chemotherapy was ceased after surgical intervention in 13 patients because of poor PS or rapid tumor growth after surgery (Table 1). The MST in the 41 patients who underwent chemotherapy was significantly longer than that of the remaining 20 patients (10 months versus 3 months, *P* = 0.006). To select those patients likely to survive intensive treatment, we analyzed several prognostic factors. Table 2 shows the correlations between several prognostic factors and patient survival. According to our data, patient age, serum CEA level, and location of tumors (data not shown) were not correlated with patient survival.

**Table 2:** Prognostic parameters in patients with unresectable advanced colorectal carcinoma

		**n**	**MST (months)**	***P***
Age (years)	≥75	22	9	0.303
	<75	39	9	
ECOG-PS	0 or 1	46	10	0.022
	2 or 3	15	5	
GPS	0 or 1	37	12	<0.001
	2	24	4	
NLR	≥5	29	4	0.002
	<5	32	13	
PNI	≥40	35	10	0.002
	<40	26	4	
CEA (ng/mL)	≥10	33	9	0.268
	<10	28	9	

CEA, carcinoembryonic antigen; ECOG-PS, Eastern Cooperative Oncology Group Performance Status; GPS, Glasgow Prognostic Score; MST, median survival time; NLR, neutrophil/lymphocyte ratio; PNI, prognostic nutritional index.

The mean levels of CRP and ALB, lymphocyte count in peripheral blood, NLR, PNI, and CEA in the 61 patients were 3.2 (range 0.01 to 13.9) mg/dL, 3.4 (range 1.8 to 4.7) g/dL, 1,322 (range 123 to 2,899)/mm^2^, 5.7 (range 1.1 to 31), 40.6 (range 19.6 to 58) and 567 (range 1.2 to 20,157) ng/mL, respectively. Fifty-three patients died of progressive disease during the follow-up period and the remaining eight were alive at May 2014. Only one patient underwent complete resection of both distant metastatic sites (lung and liver) and the primary tumor after bypass surgery followed by intensive chemotherapy, and this patient remained alive at 75 months after the initial visit to our hospital, without recurrence or additional treatment. Primary tumor resection was performed in a further two patients, one of whom died at 35 months as a result of progression of liver metastasis, and the other remained alive at 13 months, with chemotherapy. Conversion chemotherapy was therefore performed in 4.9% (3/61) of patients with initially unresectable locally advanced colorectal cancer.

**Figure 1:**
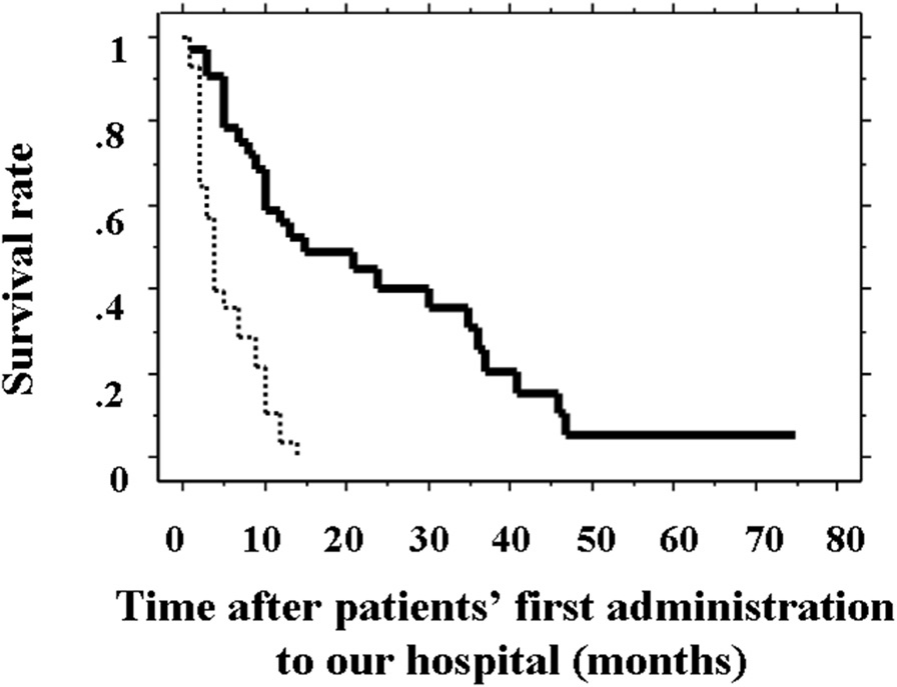
**Survival curves of patients with a low- and high- score groups.** The survival curve of 33 patients with low scores (score 0 and +1; solid line) was significantly higher than that for 28 patients with high scores (score +2, +3, and +4; dashed line; *P* < 0.001).

Table 3 indicates our scoring system. According to our scoring system, patients with abnormal PS, GPS, NLR, and PNI had a score of 4. Sixteen patients scored 0, 17 scored +1, 10 scored +2, 17 scored +3, and 1 scored +4. The 61 patients were divided into two groups based on our scoring system: a low-score group (0 and +1) and a high-score group (+2, +3, and +4). The MST of the 33 patients in the low-score group (15 months) was significantly higher than that of the 28 patients in the high-score group (4 months, *P* < 0.001; Figure 1). Three patients who underwent conversion chemotherapy were in the low-score group. Thus, these results indicate that this scoring system based on a combination of several prognostic factors may represent a useful method for selecting patients with initially unresectable advanced colorectal cancer who will survive.

**Table 3:** Scoring system

		**n**	**Score**
ECOG-PS	0 or 1	46	0
	2 or 3	15	+1
GPS	0 or 1	37	0
	2	24	+1
NLR	≥ 5	29	+1
	< 5	32	0
PNI	≥ 40	35	0
	< 40	26	+1

ECOG-PS, Eastern Cooperative Oncology Group Performance Status; GPS, Glasgow Prognostic Score; NLR, neutrophil/lymphocyte ratio; PNI, prognostic nutritional index.

## Discussion

R0 (complete resection of tumors) surgery is the only treatment associated with long-term survival in patients with advanced colorectal cancer. However, this radical surgery may have to be omitted because of numerous distant metastases or local advancement of tumors. Effective chemotherapy should be administered and palliative surgery (bypass surgery or artificial anus reconstruction) performed in such patients. Even if the tumor is deemed unresectable at the time of administration (initially unresectable colorectal cancer), complete resection of both primary and metastatic tumors can be performed after palliative surgery and intensive chemotherapy in a few patients. Such conversion therapy has been associated with favorable long-term survival in patients with initially unresectable colorectal cancer [13-15]. In the present study, we analyzed the factors used to estimate who can undergo conversion therapy in patients with locally advanced unresectable colorectal cancer who had palliative surgery or chemotherapy.

Although conversion chemotherapy has been discussed in relation to unresectable liver metastases, few studies have considered conversion chemotherapy for locally advanced colorectal cancer [16,17]. Hsu and colleagues [12] reported that aggressive chemotherapy had a survival benefit, even in patients with unresectable locally advanced colorectal cancer. They concluded that high a CEA level was a prognostic factor for poorer survival in these patients. In this study, surgical resection of primary sites, or both primary and metastatic sites, could be performed after intensive chemotherapy in 5% of patients with initially unresectable and locally advanced colorectal cancer. However, prior to administering intensive chemotherapy and performing conversion chemotherapy, it is necessary to identify those patients likely to survive. The CEA level is widely used to monitor recurrence during postoperative follow-up, and high serum CEA levels are associated with advanced tumor stage, poor prognosis, and reduced survival in patients with colorectal cancer. CEA may therefore be a good indicator of tumor progression. However, in our study we found that high serum CEA levels did not reflect survival of patients with locally advanced colorectal cancer. Also, many reports suggest that CEA levels did not correlate with tumor progression in many colorectal tumors [10,11]. Thus, we excluded the pretreatment serum CEA level from our scoring system.

CRP, serum ALB, and peripheral lymphocyte count in cancer patients have recently attracted research attention. These factors may act as indicators of inflammation, nutrition, and immunity. GPS is well known as an inflammatory and nutritional prognostic parameter in various cancers, and may be a useful marker for treatment administration in advanced cancers [18,19]. Ikuta and colleagues [19] reported that poor GPS may be a negative indicator for palliative bypass surgery in unresectable pancreatic or biliary cancers. NLR may also indicate the patient’s inflammation and immunity status. Circulating lymphocytes are known to play an important immunological role in various carcinomas [20,21]. Additionally, a strong correlation between cancer progression and lymphopenia was detected in patients with clear cell renal carcinoma [22]. Lissoni and colleagues reported that lymphocytosis occurred independently of tumor histotype and chemotherapeutic regimen in patients who achieved objective tumor regression in response to chemotherapy, and that the mean lymphocyte count was significantly increased after chemotherapy [23]. They added that the mean lymphocyte count decreased with chemotherapy in patients with tumor progression.

Furthermore, ECOG-PS and Onodera’s PNI are known nutritional markers. Although both these markers may be strong prognostic indicators, analyzing them separately may not be enough to select patients able to survive intensive treatment. We therefore advocated a new scoring system using ECOG-PS, GPS, NLR, and PNI. The MST in patients with low scores (score 0 or +1) was 15 months, which was significantly longer than that of patients with high scores (+2, +3, or +4; 4 months). Although the sample size in this study was small, the scoring system was able to predict the prognosis of patients with unresectable advanced colorectal cancer during treatment. In order to prove this fact, future large-scale study is needed.

## Conclusion

Patients with locally advanced colorectal cancer with distant metastasis who have low scores may be able to survive through intensive treatment, even if they were deemed to be unresectable at the time of pretreatment. The number of patients of our study was small, but the results from our primitive study may lead us to large-scale study in the future.

## Abbreviations

ALB: albumin; CEA: carcinoembryonic antigen; CRP: C-reactive protein; ECOG-PS: Eastern Cooperative Oncology Group Performance Status; GPS: Glasgow Prognostic Score; MST: median survival time; NLR: neutrophil/lymphocyte ratio; PNI: prognostic nutritional index; PS: performance status.

## Competing interests

The authors declare that they have no competing interests.

## Authors’ contributions

All authors read and approved the final manuscript.
